# The essential role of CCT2 in the regulation of aggrephagy

**DOI:** 10.3389/fnagi.2024.1491001

**Published:** 2024-10-16

**Authors:** Jie Luo, Ze-Sen Feng, Ji-Xin Tang

**Affiliations:** Guangdong Provincial Key Laboratory of Autophagy and Major Chronic Non-communicable Diseases, Key Laboratory of Prevention and Management of Chronic Kidney Diseases of Zhanjiang City, Institute of Nephrology, Affiliated Hospital of Guangdong Medical University, Zhanjiang, China

**Keywords:** CCT2, protein aggregate, aggrephagy, chaperonin, neurodegenerative diseases

## Abstract

Protein aggregation, a defining characteristic of numerous human diseases, poses a significant challenge to cellular health. Autophagy, an essential cellular recycling process, specifically targets and degrades these harmful protein aggregates through a specialized mechanism known as aggrephagy. However, the precise mechanisms underlying the exquisite selectivity of aggrephagy in identifying and eliminating only aggregated proteins while sparing healthy cellular components have remained enigmatic. Here, in this mini review, we highlights the essential role of CCT2, a subunit of the chaperonin TRiC complex, in regulating aggrephagy. CCT2, traditionally viewed as a molecular chaperone, has emerged as a novel autophagy receptor that specifically targets solid protein aggregates for degradation. This ubiquitination-independent mode of recognition by CCT2 expands our understanding of protein degradation pathways. The functional switch of CCT2 from a chaperone to an autophagy receptor underscores its dynamic nature and ability to adapt to cellular stress. The selectivity of CCT2-mediated aggrephagy for solid aggregates has implications for neurodegenerative diseases. Further research is warranted to explore the therapeutic potential of enhancing CCT2-mediated aggrephagy in such diseases.

## 1 Introduction

Protein aggregation, the abnormal accumulation of misfolded or unfolded proteins, is a ubiquitous phenomenon associated with numerous human pathologies, including neurodegenerative diseases, metabolic disorders, and cancer (Louros et al., 2023; Wen et al., 2023). These aggregations often lead to cellular dysfunction and, ultimately, tissue damage and organ failure. To combat this threat, cells have evolved intricate mechanisms to maintain protein homeostasis (proteostasis), including the molecular chaperones, ubiquitin-proteasome system (UPS) and autophagy (Lv et al., 2023; Klaips et al., 2018; Hipp et al., 2019). Among these, autophagy, particularly aggrephagy—a subtype of autophagy specifically targeting protein aggregates—has garnered significant attention due to its pivotal role in the clearing toxic protein aggregates (Bauer et al., 2023).

Chaperonin Containing TCP-1 (CCT), also known as TRiC (TCP-1 Ring Complex), is a multi-subunit protein complex essential for the folding of approximately 10% of cytosolic proteins (Gestaut et al., 2022; Betancourt Moreira et al., 2023; Jin et al., 2019). CCT is composed of eight distinct subunits (CCT1-8), each playing a critical role in maintaining the structural integrity of nascent polypeptides. Among these, CCT2, a subunit of CCT, has recently emerged as a novel player in the regulation of aggrephagy, shedding light on the intricate interplay between protein folding and degradation (Ma et al., 2022a; Strzyz, 2022; Zhang and Klionsky, 2022).

This mini review outlines CCT2’s dual roles: as a molecular chaperone crucial for protein folding and homeostasis, and recently, as an autophagy receptor in aggrephagy, degrading solid protein aggregates to maintain proteostasis (Ma et al., 2022a; Strzyz, 2022; Zhang and Klionsky, 2022; Ma et al., 2022b; Chen and Zhang, 2022). We detail CCT2’s mechanisms in aggrephagy, emphasizing its interplay with cellular clearance machinery. We also discuss CCT2’s potential as a therapeutic target for diseases linked to protein misfolding, notably neurodegeneration. Finally, we address open questions and propose future research to deepen our understanding of CCT2’s multifaceted contributions, with implications for developing novel therapies to alleviate related pathologies and improve patient outcomes.

## 2 Previous insights into CCT2: the subunit of the chaperonin TRiC

For decades, CCT2 has been recognized as an integral part of the CCT/TRiC complex, facilitating the correct folding of a wide range of proteins, including actin, tubulin, and various enzymes. This role is crucial for maintaining cellular protein homeostasis and ensuring proper cellular function. Mutations in CCT2 or other CCT subunits have been linked to various human diseases, including Leber congenital amaurosis, underscoring the importance of this complex in cellular physiology (Minegishi et al., 2016; Roy et al., 2023; Minegishi et al., 2018; Suga et al., 2024).

However, the function of CCT2, and CCT in general, has been primarily confined to its role as a molecular chaperone (Macario and Conway de Macario, 2022; Zhao et al., 2024; Guest et al., 2015). Recent advancements, however, have challenged this notion, revealing that CCT2 possesses additional, non-canonical functions that extend beyond protein folding. One such function is its emerging role as an autophagy receptor in aggrephagy (Ma et al., 2022a).

## 3 CCT2 as an autophagy receptor in aggrephagy

The discovery that CCT2 serves as an autophagy receptor for the clearance of solid protein aggregates represents a paradigm shift in our understanding of protein degradation pathways (Ma et al., 2022a; Strzyz, 2022; Zhang and Klionsky, 2022; Ma et al., 2022b). This finding not only expands the repertoire of autophagy receptors but also highlights the versatility of CCT2 in maintaining cellular protein homeostasis.

### 3.1 The molecular mechanism of CCT2-mediated aggrephagy

CCT2 mediates aggrephagy through a unique mechanism that is distinct from canonical autophagy receptors such as p62, NBR1, and TAX1BP1 (Ma et al., 2022a). These receptors typically recognize ubiquitinated cargo and facilitate their sequestration into autophagosomes via interactions with ATG8 family proteins, including LC3. In contrast, CCT2 interacts with aggregation-prone proteins independently of their ubiquitination status, indicating a ubiquitination-independent mode of recognition.

CCT2 achieves this by harboring a non-canonical LC3-interacting region (LIR motif), termed VLIR, which allows it to directly bind ATG8s, including LC3. This interaction is crucial for targeting protein aggregates to autophagosomes for degradation. Importantly, the VLIR motif is exposed only when CCT2 exists as a monomer, a conformational change that occurs upon the accumulation of aggregation-prone proteins. This functional switch from a chaperone subunit to an autophagy receptor underscores the dynamic nature of CCT2’s function and its ability to adapt to cellular stress.

### 3.2 Independence from canonical autophagy receptors and chaperone-mediated autophagy

CCT2-mediated aggrephagy operates independently of canonical autophagy receptors, including p62, NBR1, and TAX1BP1 (Turco et al., 2021; Sarraf et al., 2020; Da Silva et al., 2024). Even in the absence of these receptors, CCT2 efficiently promotes the clearance of solid protein aggregates, highlighting its unique role in this process. Furthermore, CCT2-mediated aggrephagy does not rely on chaperone-mediated autophagy (CMA), another protein degradation pathway involving the lysosome (Kaushik and Cuervo, 2018; Yang et al., 2019; Dong et al., 2021; Bourdenx et al., 2021). Knockdown of key CMA components does not affect CCT2’s ability to mediate aggrephagy, further underscoring its autonomy.

### 3.3 Selectivity for solid protein aggregates

The transition of misfolded proteins into solid protein aggregates undergoes a crucial liquid–liquid phase separation stage (Babinchak and Surewicz, 2020; Patel et al., 2015; Ray et al., 2020; Wegmann et al., 2018). Previous studies demonstrated that autophagy selectively degrades liquid protein aggregates, whereas solid protein aggregates were deemed less amenable to autophagic clearance, or even intractable, ultimately sequestered within cells as inclusions to mitigate cellular damage (Yamasaki et al., 2020; Zhang et al., 2018). Ma et al. (2022a) addressed this paradigm by engineering a cellular model that recapitulates the liquid-to-solid transition of protein aggregates. Employing photobleaching techniques and genetic manipulations, they discovered that CCT2 and ubiquitin-binding receptors (p62, NBR1, TAX1BP1) differentially target aggregates of varying fluidity for degradation. Notably, ubiquitin-binding receptors preferentially engage with more fluid protein aggregates (liquid-like), whereas CCT2 exhibits a proclivity for less mobile, solid aggregates, thereby mediating their autophagic clearance. This finding sheds light on the intricate machinery that governs the disposal of diverse protein aggregates within cells. The ability of CCT2 to specifically target solid aggregates suggests that it may play a unique role in diseases characterized by the accumulation of insoluble protein aggregates, such as neurodegenerative diseases.

A recent study highlights the crucial role of CCT2 in neurodegenerative diseases, particularly exploring its significance in Alzheimer’s disease (AD) via multi-omics analysis (Ma et al., 2023). Through bioinformatics analysis, CCT2 downregulation was observed in AD patients, linked to impaired autophagic clearance of *β*-amyloid. Genes associated with CCT2-high status implicated protein folding, autophagy, and mRNA stability, suggesting CCT2’s positive correlation with autophagy pathways and negative impact on neuronal death. A predictive model with 13 key genes, including CCT2, accurately forecasts AD occurrence (AUC = 0.9671), offering a potential tool for AD biomarker discovery. Furthermore, the study predicts microRNAs and small molecule drugs targeting CCT2-related genes, suggesting low CCT2 expression contributes to autophagy suppression in AD, thereby elucidating its pathogenesis and revealing novel therapeutic targets and inhibitors.

## 4 The functional switch of CCT2: from chaperone to autophagy receptor

The functional switch of CCT2 from a chaperone subunit to an autophagy receptor represents a fascinating example of cellular adaptation to stress (Ma et al., 2022a; Strzyz, 2022; Zhang and Klionsky, 2022; Ma et al., 2022b). Under normal conditions, CCT2 is part of the CCT/TRiC complex, where it contributes to protein folding. However, upon the accumulation of aggregation-prone proteins, CCT2 dissociates from the complex, adopting a monomeric form that exposes the VLIR motif. This conformational change enables CCT2 to bind ATG8s and function as an autophagy receptor, thereby targeting protein aggregates for degradation.

**Figure 1:**
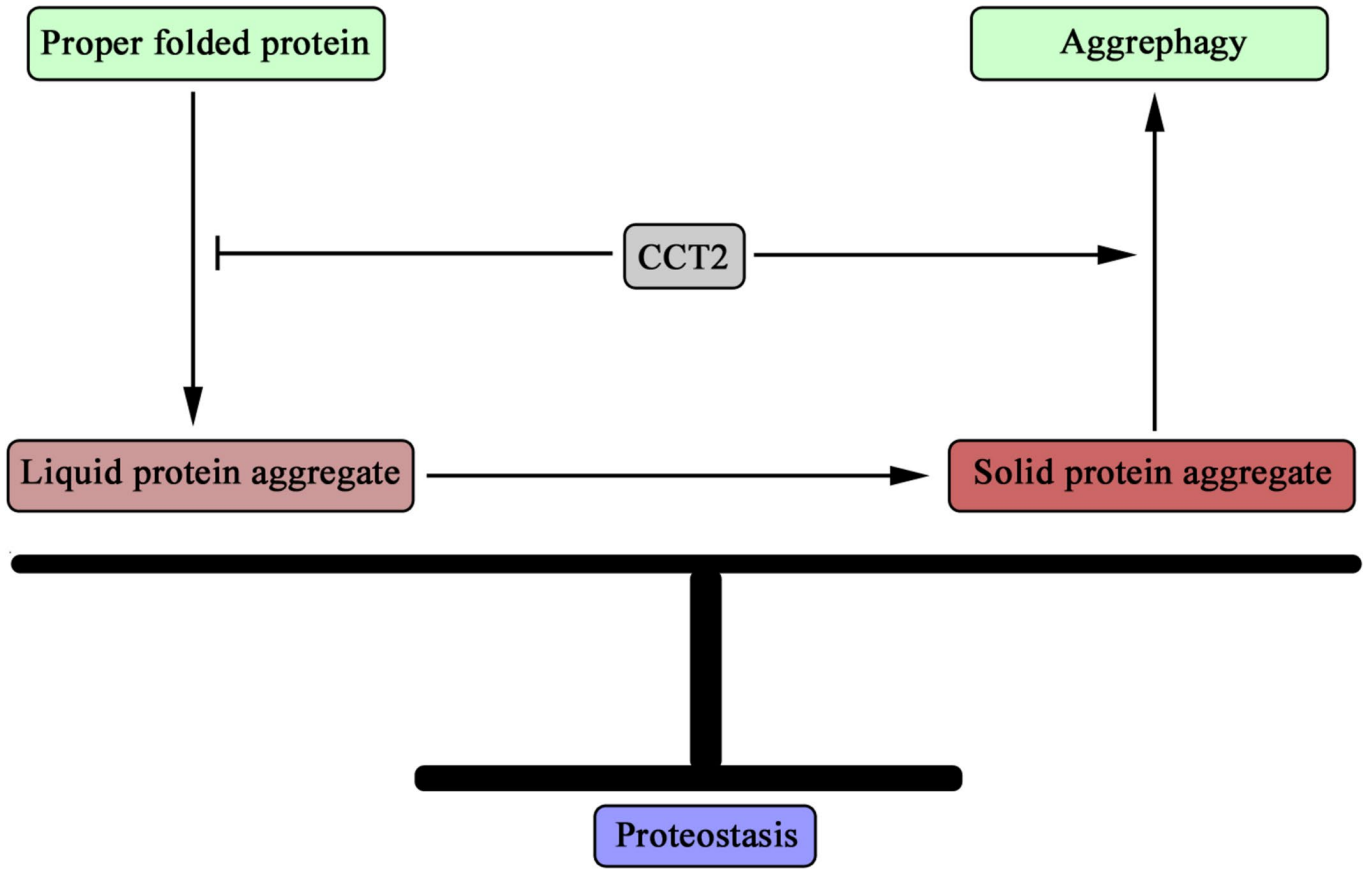
Dual role of CCT2 in maintaining proteostasis. CCT2 can act as a molecular chaperone to help proteins maintain the correct structure, thereby inhibiting the accumulation of misfolded proteins. On the other hand, when misfolded proteins are increased in cells and the liquid protein aggregates are transformed into solid protein aggregates, CCT2 plays the function of autophagy receptor, mediating the degradation of solid protein aggregates through autophagy pathway, that is, promoting the process of aggregate autophagy. Through these two roles, CCT2 plays an important role in the maintenance of cellular protein homeostasis.

Through functional transitions, CCT2 plays dual roles in maintaining proteostasis (Figure 1). In the early stages of protein homeostasis imbalance, CCT2 collaborates with other CCT subunits as a molecular chaperone, facilitating the correct folding of proteins and thereby safeguarding cellular protein stability. As the imbalance intensifies, leading to the formation of protein aggregates, particularly solid aggregates within cells, CCT2 dissociates from the CCT complex and assumes a distinct role as an autophagy receptor. By binding to these solid aggregates and directing them toward autophagosomes, CCT2 orchestrates their degradation via the autophagic pathway, further reinforcing cellular proteostasis. Consequently, CCT2 exerts a bifunctional role in preserving proteostasis, ensuring that proteins remain in a suitable and non-deleterious state within cells, thereby underlining its pivotal role in sustaining cellular health.

## 5 Discussion

The identification of CCT2 as an autophagy receptor for the clearance of solid protein aggregates represents a significant advancement in the field of autophagy research. This finding challenges the traditional view of CCT2 as a purely chaperone subunit and underscores the multifaceted nature of its function. Moreover, the revelation of a ubiquitination-independent mode of aggrephagy adds another layer of complexity to our understanding of protein degradation pathways.

The selective degradation of solid protein aggregates by CCT2 has important implications for the treatment of diseases characterized by the accumulation of insoluble protein deposits, such as Alzheimer’s disease, Parkinson’s disease, and Huntington’s disease (Erkkinen et al., 2018; Fernández-Ruiz et al., 2015; Sujkowski et al., 2022). By enhancing CCT2-mediated aggrephagy, it may be possible to reduce the burden of toxic protein aggregates and slow disease progression. However, further research is needed to fully elucidate the role of CCT2 in these diseases and to develop targeted therapeutic strategies.

While the current study provides compelling evidence for the role of CCT2 in aggrephagy, several limitations remain. Firstly, the majority of experiments were conducted *in vitro*, using cell culture models. While these experiments have provided valuable insights, *in vivo* validation in animal models is crucial to confirm the physiological relevance of CCT2’s function. For example, CCT2 conditional knockout mice could be constructed and combined with the disease model to study the impact of CCT2 loss on the disease process, so as to elucidate the role of CCT2 and its impact on the disease progression *in vivo*. Furthermore, although CCT2 is proved to be able to degrade solid protein aggregates through autophagy, a process that is noteworthy for its independence from ubiquitination of the substrates. Nevertheless, the precise molecular mechanisms underlying CCT2’s recognition of solid protein aggregates, as well as its discrimination between liquid and solid protein aggregates, remain elusive. The specific molecular pathways employed by CCT2 to achieve this selective degradation are yet to be fully understood.

Additionally, the precise molecular mechanisms underlying the functional switch of CCT2 from a chaperone subunit to an autophagy receptor are still unclear. Understanding the triggers and regulators of this switch will provide valuable insights into the regulation of aggrephagy and may lead to the development of novel therapeutic strategies. Finally, while mutations in CCT2 have been linked to congenital cataracts, the potential link between these mutations and defects in aggrephagy remains unexplored. Further research is needed to determine whether CCT2-mediated aggrephagy plays a role in the pathogenesis of this and other CCT-related diseases.

In conclusion, the discovery of CCT2 as an autophagy receptor for the clearance of solid protein aggregates represents a significant breakthrough in our understanding of protein degradation pathways. This finding not only expands the repertoire of autophagy receptors but also highlights the dynamic nature of CCT2’s function and its ability to adapt to cellular stress. Future research will undoubtedly uncover additional roles for CCT2 in proteostasis and disease pathogenesis, leading to the development of novel therapeutic strategies for a wide range of human diseases.
